# Effect of perfluoroalkyl exposure in pregnancy and infancy on intrauterine and childhood growth and anthropometry. Sub study from COPSAC2010 birth cohort

**DOI:** 10.1016/j.ebiom.2022.104236

**Published:** 2022-08-26

**Authors:** Astrid Sevelsted, Gözde Gürdeniz, Daniela Rago, Casper-Emil Tingskov Pedersen, Jessica A. Lasky-Su, Antonio Checa, Pei Zhang, Craig E. Wheelock, Stine S. Normann, David M. Kristensen, Morten Arendt Rasmussen, Jörg Schullehner, Kalliroi Sdougkou, Jonathan W. Martin, Jakob Stokholm, Klaus Bønnelykke, Hans Bisgaard, Bo Chawes

**Affiliations:** a COPSAC, Copenhagen Prospective Studies on Asthma in Childhood, Herlev and Gentofte Hospital, University of Copenhagen, Copenhagen, Denmark; b Brigham and Women's Hospital and Harvard Medical School, Boston, MA 02115, United States; c Unit of Integrative Metabolomics, Institute of Environmental Medicine, Karolinska Institutet, Stockholm 171-77, Sweden; d Department of Respiratory Medicine and Allergy, Karolinska University Hospital, Stockholm 141-86, Sweden; e Gunma University Initiative for Advanced Research (GIAR), Gunma University, 3-39-22 Showa-machi, Maebashi, Gunma 371-8511, Japan; f Department of Neurology, Danish Headache Center, Rigshospitalet-Glostrup, University of Copenhagen, Copenhagen, Denmark; g Department of Biology, University of Copenhagen, Copenhagen, Denmark; h Department of Groundwater and Quaternary Geology Mapping, Geological Survey of Denmark and Greenland, Aarhus, Denmark; i Research Unit for Environment, Work and Health, Department of Public Health, Aarhus University, Aarhus, Denmark; j Science for Life Laboratory, Department of Environmental Science, Stockholm University, Stockholm 114 18, Sweden

**Keywords:** MeSH, PFOS, PFOA, Metabolomics, Xenobiotics, Child, Mother-child cohort, BMI, Growth, Lactocyl Ceramides, Molecular epidemiology, BMI, body mass index, COPSAC, Copenhagen Prospective Studies on Asthma in Childhood, DXA, dual-energy x-ray absorptiometry, FDR, false discovery rate, FLG, filaggrin, HbA1c, hemoglobin A1c, HDL, high density lipoprotein, LacCer, lactoceramides, LDL, low density lipoprotein, PFOA, perfluorooctanoic acid, PFOS, perfluorooctane sulfonic acid, PCA, principal component analysis, UPLC-MS/MS, Ultra Performance Liquid Chromatography/Mass Spectrometry, UPLC-TQD-MS/MS, Ultra Performance Liquid Chromatography Triple Quadrupole Detection/Mass Spectrometry, PFAS, Per- and polyfluoroalkyl substances (including PFOS and PFOA), CMPF, 3-carboxy-4-methyl-5-propyl-2-furanpropanoic acid (biomarker for fish intake)

## Abstract

**Background:**

Perfluoroalkyl substances PFOS and PFOA are persistent and bioaccumulative exogenous chemicals in the human body with a range of suspected negative health effects. It is hypothesised that exposure during prenatal and early postnatal life might have particularly detrimental effects on intrauterine and childhood growth. In a Danish longitudinal mother-child cohort we investigate effect of PFOS and PFOA in pregnancy and infancy on intrauterine and childhood growth and anthropometry.

**Methods:**

COPSAC_2010_ is an ongoing population based mother-child cohort of 738 pregnant women and their children followed from 24 week gestation with longitudinal deep clinical phenotyping until age 10 years. In this observational cohort sub study plasma PFOS and PFOA concentrations were semi-quantified by untargeted metabolomics in the mothers at week 24 and 1 week postpartum and in the children at ages 6 and 18 months and calibrated using a targeted pipeline. We examined associations to intrauterine and childhood growth and anthropometry, including interactions with child sex. Untargeted and targeted blood metabolomics profiles were integrated to investigate underlying mechanisms.

**Findings:**

Pregnancy plasma PFOA concentrations were associated with lower birth size −0.19 [−0.33; −0.05] BMI z-score per 1-ng/mL and increased childhood height (z-scored) at age 6: 0.18 [0.05; 0.31], but there was no association between childs’ own infancy plasma PFOA concentration and height. Pregnancy plasma PFOS concentrations were also associated with lower birth BMI (−0.04 [−0.08; −0.01]), but in childhood pregnancy plasma PFOS concentration interacted with child sex on BMI and fat percentage at 6 years with negative associations in girls and positive in boys. The effect of maternal plasma PFOS concentration on lower girl BMI was borderline mediated through increasing child plasma lactosyl-ceramide levels (*p*-mediation=0.08). Similarly the effect of maternal plasma PFOS concentration on higher boy fat percentage was borderline mediated through increasing child plasma lactosyl-ceramide levels (*p*-mediation=0.07). Infancy concentrations of plasma PFOS associated with lower height in childhood, −0.06 z-score at age 6 [−0.19; −0.03].

**Interpretation:**

Higher PFOS and PFOA plasma concentrations during pregnancy had detrimental effects on fetal growth. The effects on childhood growth were not similar as PFOA increased child height, opposite of PFOS in multipollutant models suggesting a differing fetal programming effect. Sex specific growth effects were borderline mediated through an altered lactosyl-ceramide metabolism, proposing a possible mechanism of PFOS that has long-lasting health consequences in this observational study.

**Funding:**

All funding received by COPSAC are listed on www.copsac.com. The Lundbeck Foundation (Grant no R16-A1694); The Novo Nordic Foundation (Grant nos NNF20OC0061029, NNF170C0025014, NNF180C0031764) The Ministry of Health (Grant no 903516); Danish Council for Strategic Research (Grant no 0603-00280B) and The Capital Region Research Foundation have provided core support to the COPSAC research center. Effort from JALS is supported by R01HL123915, R01HL141826, and R01HL155742 from NIH/NHLBI. CEW was supported by the Swedish Heart Lung Foundation (HLF 20180290, HLF 20200693). BC has received funding for this project from the European Research Council (ERC) under the European Union's Horizon 2020 research and innovation programme (grant agreement No. 946228). The funding agencies did not have any role in design and conduct of the study; collection, management, and interpretation of the data; or preparation, review, or approval of the manuscript.

**Research in context**

**Evidence before this study**

Perfluoroalkyl substances are persistent and bioaccumulative exogenous chemicals in the human body with a range of suspected negative health effects. It is hypothesised that exposure during prenatal and early postnatal life might have particularly detrimental growth effects. We did a systematic search using keywords: Perfluorooctane sulfonate (PFOS); perfluorooctanoic acid (PFOA); child; mother; longitudinal; BMI; growth; Denmark. Previous studies primarily linked pregnancy exposure to increased risk of child obesity.

**Added value of this study**

In a longitudinal mother-child cohort we detected plasma PFOS and PFOA in all samples (>600) of both mothers and offspring. Maternal plasma PFOA and PFOS concentrations were associated with reduced intrauterine growth. Maternal plasma PFOA concentrations associated to taller children. Maternal plasma PFOS concentrations interacted with child sex on 6 years BMI which was lower in girls and increased in boys, these effects were borderline mediated through an altered child lactosyl-ceramide metabolism.

**Implications of all the available evidence**

Our data showed opposing effect of maternal plasma PFOS and PFOA concentrations on child height. Maternal plasma PFOS concentration interacted with child sex, and we found a possible mechanism through an altered child lactosyl-ceramide metabolism which interacted with child sex.

Alt-text: Unlabelled box

## Introduction

Environmental exposure to a broader range and higher levels of xenobiotics, i.e., chemicals found in an organism that are extrinsic to the normal metabolism has increased in recent generations.^1^ Populations are increasingly exposed to xenobiotics from the food we eat, the water we drink, the clothes we wear and the utensils we use.^2^

Among xenobiotics, persistent and bioaccumulative chemicals such as perfluoroalkyl substances that include perfluorooctane sulfonate (PFOS) and perfluorooctanoic acid (PFOA) have created particular concern with regard to early life development.^3^^,^^4^ Typical routes of exposure are through drinking water, diet, consumer products and these compounds may affect human development already in fetal life, since they have the ability to pass the placental barrier, and can be found in amniotic fluid and umbilical cord blood of the newborn.^5^^,^^6^ PFOS and PFOA have potential endocrine-disruptive abilities, and are suspected to influence fetal programming of the metabolism.^7^^,^^8^

Previous studies have primarily linked maternal plasma concentrations of both PFOS and PFOA to offspring obesity traits.^8^^,^^9^ Some studies have shown differing effect of maternal plasma PFOS and PFOA concentrations linking pregnancy plasma PFOS concentrations to lower growth and PFOA to higher adiposity traits,^10^ especially when applying multipollutant models, since PFAS are highly correlated but may show different growth effect in the child.

This study aims to investigate the population based COPSAC2010 birth cohort for the longitudinal effect of repetitive measures of plasma concentrations of both PFOS and PFOA during pregnancy and infancy on intrauterine and childhood growth and anthropometry during the first 10 years of life. Underlying mechanisms were investigated by integrating untargeted and targeted blood metabolomics profiles.

## Methods

This study is part of the ongoing longitudinal unselected mother-child cohort, the Copenhagen Prospective Studies of Asthma in Childhood 2010 (COPSAC-2010). Pregnant women from Zealand, Denmark recruited from the monthly surveillance of reimbursement to general practitioners for the standard pregnancy visit were invited to participate in the study with main focus on development of asthma during 2008–2010. A total of 738 women were enrolled at pregnancy week 24. The pregnant women and their children, including five twin pairs, attended 14 scheduled clinical visits plus acute care visits in the first 10 years of life.

### Untargeted metabolomics profiles

Untargeted ultra-performance liquid chromatography tandem mass spectrometry (UPLC-MS/MS) plasma metabolomics profiling of the pregnant mothers at 24 week and 1 week postpartum and of the children at age 6 months, 18 months and 6 years was performed using untargeted plasma metabolomic profiling including relative abundances of PFOS and PFOA from the HD4 platform Metabolon, Inc. (NC, USA) as described previously.^11^

### Calibration of semi-quantitative PFOS and PFOA

A selection of 48 samples representing the range of untargeted PFAS concentrations in the study were absolute quantified for PFOS and PFOA using a targeted method for perfluoroalkyl acids modified from Glynn et al.^12^

Briefly, an aliquot of 0.2 mL of plasma was placed in a 15 mL conical polypropylene centrifuge tube and spiked at a concentration of 1 ng/ml with 13 labeled internal standards (MPFAC-C-ES, Wellington Laboratories, Wellington Laboratories). The plasma was extracted by protein precipitation with 4 mL of acetonitrile (ACN), followed by sonication at room temperature for 10 min and centrifugation at 2000 rpm for 5 min. The supernatant was transferred to a new 15 mL polypropylene tube and concentrated with nitrogen gas at 30 °C to a volume of 0.2 mL. The extract was then diluted to 1 mL to a 50:50 methanol:water solvent composition before undergoing dispersive clean-up. For the clean-up, the extract was transferred to a 1.5 mL tube containing 0.025 g of bulk graphitized carbon (Supelclean ENVI-Carb, Sigma Aldrich), that had been acidified with 50 µL of glacial acetic acid and vortexed for 10 s. The sample was then centrifuged for 10 min at 14,000 rpm and the top 0.5 mL was filtered with 0.2 μm nylon centrifuge filters (Thermo Scientific™ 750 μL Nonsterile Micro-Centrifugal Filters). An aliquot of 0.2 ml of the filtered extract was transferred to a glass auto-sampler vial for analysis.

Analysis was performed by ultra-high pressure liquid chromatography (HPLC, Ultimate 3000) coupled to a HRMS Q Exactive Orbitrap HF-X (ThermoFisher Scientific, Waltham, MA, USA) with electrospray ionization (ESI). The mass spectrometer was operated in negative ESI mode and alternated between a full MS scan (90 to 1000 m/z, 120,000 resolution FWHM at 200 m/z) and four MS2 data-independent acquisition (DIA) scans (30,000 resolution) with variable m/z precursor windows. A 10 μL sample volume was injected onto an ACQUITY UPLC BEH C18 analytical column (130Å, 1.7 μm, 2.1 mm × 100 mm, Waters) equipped with a ACQUITY BEH C18 1.7 µM VANGUARD Pre-Column at 40°C. Upstream of the injector, one ACQUITY UPLC BEH C18 analytical column (130Å, 1.7 μm, 3 mm × 30 mm, Waters) was in place to separate instrumental background PFOS and PFOA from the analytes in the sample. A binary gradient elution was used, including (A) 2 mM ammonium acetate, and (B) 100% methanol at 0.35 mL/min.

The raw data were extracted and the peak area of the molecular ions of the analytes were integrated using Xcalibur software (Thermo Scientific, version 4.1). Quantification was performed using an external solvent-based calibration curve with the internal standard method.

When extrapolating the semi-quantitative measures to absolute values, several child plasma PFOA concentration were extrapolated to negative values. For this reason child plasma PFOA concentrations were offset by 0.8 in all analyses where log transform was used.

Pregnancy plasma concentrations of PFOS and PFOA was calculated as the mean values of the two maternal measurements (pregnancy week 24 and 1 week postpartum) and early life exposure as the mean values of the child age 6 and 18 months measurements.

### Targeted sphingolipids

A total of 10 different lactosyl-ceramide species (LacCers) were quantified or pseudo-quantified by Ultra Performance Liquid Chromatography Triple Quadrupole Detection/Mass Spectrometry (Waters Xexo TQ-S) in plasma samples at age 6 years as described previously.^13^

### Intrauterine and childhood growth and anthropometry endpoints

Fetal growth was estimated from ultrasound scans at pregnancy week 20 (Hadlock estimated weight) and anthropometric measurements at 1 week postpartum. In addition, gestational age, birth weight, BMI and Skjerven percentile (birth weight adjusted for gestational age and sex).^14^

Childhood growth was assessed measuring weight and height and calculating BMI at all clinical study visits. Age at adiposity rebound was estimated from repeated growth measurements.^15^^,^^16^ Weight, height and BMI are z-scored to WHO reference population.^17^

Body composition was determined by Dual-energy X-ray Absorptiometry (DXA) scans,^18^ and cholesterol, low-density lipoprotein cholesterol (LDL-C), high-density lipoprotein cholesterol (HDL-C) and triglyceride were measured in venous non-fasting serum samples at age 6 years.

Bioelectric impedance assessment was performed at age 10. Fat free mass index (FFMI) calculated as Fat Free mass(kg)/heightsquared(m2), and Fat Mass Index (FMI) as Fat Mass(kg)/heightsquared(m2).^19^

### Covariates

Detection of PFOS and PFOA in drinking water, social circumstances, race, urban/rural living, family income, fish oil intervention, maternal: pre-pregnancy BMI, education, smoking, semi quantified maternal CMPF from the metabolome (a biomarker for fish intake) and maternal filaggrin mutation status (FLG) were selected to be analysed as relevant environmental sources/modifiers of maternal concentrations. Social circumstances were defined from principal component 1 of a principal component analysis (PCA) of variables mothers age, education, and household income explaining 56% of the variation. These variables were obtained during personal interviews in pregnancy or around the child's 2 year birthday for social circumstances.

We identified the geographical coordinates of the addresses of the cohort at birth. Based on the coordinates we inferred both the urban/rural axis from satellite images as described earlier^20^ as well as the drinking water exposure to PFOS and PFOA, for households which had water access from a public supply with a PFOS or PFOA measurement from the national monitoring programme, utilizing a spatial model of Danish water supply systems^21^ (see details in online supplement).

### Statistics

Determinants and growth effects of PFOS and PFOA exposure were investigated individually. Changes over time were investigated with geometric means at different time points. Correlations between measurements at different time points were investigated with Spearman correlation coefficients.

Geometric mean of maternal concentrations were plotted against parity and log-linear associations between maternal concentrations and parity were calculated for inference. Child concentrations were log-linear associated with duration of breastfeeding (limited to maximum 18 months) and investigated for interaction with maternal concentrations in tertiles.

Environmental determinants of maternal concentrations (log—transformed) were investigated in log-linear regression models adjusted for parity.

Associations between PFOS and PFOA plasma concentrations and growth outcomes were investigated in linear models using continuous non-transformed levels. Twins are excluded from all growth outcomes. All estimates are given as effect per 1-ng/mL. In figures all outcomes are internally z-scored for visual inspection. In tables all outcomes are on their original scale. All estimates of maternal concentrations are adjusted for parity, race, maternal social circumstances, maternal pre-pregnancy BMI, maternal height, maternal biomarker for fish intake, pregnancy fish oil supplementation RCT,^11^ birth address urbanicity, and child concentrations additionally adjusted for breastfeeding duration. In multipollutant models the results are further adjusted for the concentration of the other PFAS. Twins are excluded from all growth related outcomes. Sex interactions were investigated as interaction term between child sex and PFAS concentration. No multiple test corrections were performed for the growth outcomes.

Association between geometric mean of maternal plasma PFOS and PFOA concentration and untargeted metabolic profiles in the child measured at 6 months, 18 months and 6 years separately were investigated by linear regression. The metabolite levels were log—transformed prior to analysis. The linear models were adjusted for parity, race, maternal social circumstances, maternal pre-pregnancy BMI, maternal height, maternal biomarker for fish intake, pregnancy fish oil supplementation, birth address urbanicity, and breastfeeding duration. Multiple comparisons for metabolites were corrected for FDR using the Benjamini–Hochberg method.^22^ The relationship between maternal plasma PFOS concentration and child targeted plasma LacCers at 6 years of age was assessed with the same statistical approach.

For associations between maternal concentrations and child outcomes we sought mediation by the first component of all the correlated LacCers in a principal component analyses. Mediation analyses were done using the “mediation” package from R where a set of linear regression models were fitted and then the estimates of “mediation effects” were computed from the fitted models.^23^

Inclusion criteria was available data. Participants with missing data on either exposure (Table 1), outcomes or covariates (N=21) were excluded from analysis. No data are imputed. Sample size is based on feasibility and availability for all analyses. All analyses were done using R statistical software.

**Table 1:** Cohort baseline demographics.

	*N*	Prevalence (%)mean (SD)median [IQR]
Maternal age (mean (SD))	700	32.28 (4.36)
Maternal BMI (mean (SD))	700	24.55 (4.39)
Maternal smoking (%)	700	25 (3.6%)
Maternal FLG risk^a^ (%)	682	31 (4.5%)
Fish intake biomarker cmpf scaled (median [IQR])	686	−0.23 [−0.70, 0.45]
Maternal education (%)	700	
High		205 (29.3%)
Intermediate		444 (63.4%)
Low		51 (7.3%)
Family income, DKR (%)	700	
<400k		60 (8.6%)
400–600k		131 (18.7%)
600–800k		245 (35.0%)
800k-1 million		136 (19.4%)
>1 million		128 (18.3%)
Parity (%)	700	
1st		323 (46.1%)
2nd		267 (38.1%)
3rd+		110 (15.7%)
Drinking water >LoD^b^ (%)	520	78 (16%) / 3 (0.5%)
Land cover (%)	686	
Rural		188 (27.4%)
Intermediate		191 (27.8%)
Urban		307 (44.8%)
Race, european descent (%)	700	670 (95.7%)
Fishoil intervention group (%)	698	347 (49.7%)
Breastfeed duration, weeks (median [IQR])	692	33.57 [21.57, 46.00]

a Maternal filaggrin risk is based on SNPs: rs138726443; rs150597413; rs61816761.

b Prevalence of drinking water detection is reported for each compound, PFOA first.

### Ethics

The clinical investigations of the children and collection of biobank materials have been approved by the local Committee on Health Research Ethics (H-B-2008-093) and the Danish Data Protection Agency, ensuring that all personal data are handled according to GDPR standards and Danish law. All participating parents/caregivers have provided verbal and written informed consent for the participation of their children and use of the biobank samples for metabolomics research, genetics and other measurements in the project. Participants can withdraw that consent at any time and without any further explanation.

### Role of funders

All funding received by COPSAC are listed on www.copsac.com. The Lundbeck Foundation (Grant no R16-A1694); The Novo Nordic Foundation (Grant nos NNF20OC0061029, NNF170C0025014, NNF180C0031764) The Ministry of Health (Grant no 903516); Danish Council for Strategic Research (Grant no 0603-00280B) and The Capital Region Research Foundation have provided core support to the COPSAC research center. Effort from JALS is supported by R01HL123915, R01HL141826, and R01HL155742 from NIH/NHLBI. CEW was supported by the Swedish Heart Lung Foundation (HLF 20180290, HLF 20200693). BC has received funding for this project from the European Research Council (ERC) under the European Union's Horizon 2020 research and innovation programme (grant agreement No. 946228). The funding agencies did not have any role in design and conduct of the study; collection, management, and interpretation of the data; or preparation, review, or approval of the manuscript.

## Results

The COPSAC2010 cohort of 738 pregnant women (5 twin pairs) were recruited in 2009–2011. 43 children were excluded before birth, baseline demographics of the 700 children are presented in Table 1.

At pregnancy week 24 and 1 week postpartum, a total of 727 and 684 women respectively, had a valid measurement of both PFOS and PFOA plasma concentrations. In the children at age 6 and 18 months, 602 and 606 had a valid measurement, respectively. Both PFOS and PFOA were consistent within individuals with correlation coefficients of *R*=0.9 for both compounds in the two maternal measurements and similarly in the two measurements in the children with *R*=0.6 (PFOS) and *R*=0.7 (PFOA). Therefore, we used the average of the two maternal measurements as surrogate for the exposure of PFOS and PFOA during pregnancy, and correspondingly, we used an average of the child measurements at 6 and 18 months as surrogate for early life exposure.

Among the 675 women, who had two samples measured for PFOS and PFOA at pregnancy week 24 and 1 week postpartum, the pregnancy plasma concentrations of PFOS and PFOA was median [IQR] 6.24 ng/mL [4.96–7.73] and 1.08 ng/mL [0.78–1.47], respectively. Among the 533 children, who had two samples measured for at age 6 or 18 months, the early life plasma concentrations of PFOS and PFOA was 5.29 ng/mL [4.05–6.94] and 2.33 ng/mL [1.40–3.56], respectively (Table 2).

**Table 2:** Maternal and child levels of PFOS and PFOA.

	*N*	Median [IQR]	*N* both^a^	Median [IQR]
**Perfluorooctanesulfonate (PFOS) ng/mL**				
Maternal w 24 gestation	727	7.37 [6.01–9.09]	675	6.24 [4.96–7.73]
Maternal wk 1 postpartum	684	5.02 [3.91–6.39]		
Child 6 mth	602	4.95 [3.71–6.61]	533	5.29 [4.05–6.94]
Child 18 mth	606	5.29 [4.00–7.31]		
**Perfluorooctanoate (PFOA) ng/mL**				
Maternal w 24 gestation	727	1.22 [0.86–1.68]	675	1.08 [0.78–1.47]
Maternal wk 1 postpartum	684	0.95 [0.70–1.28]		
Child 6 mth	602	2.43 [1.10–4.06]	533	2.33 [1.40–3.56]
Child 18 mth	606	2.13 [1.34–3.06]		

a In analyses we use the mean of maternal (and child) measurements. Only persons with two measurements are included in the “N both”.

The correlation between PFOS and PFOA concentrations was 0.52 in the pregnancy measurements and 0.63 in child measurements.

### Mother-child transfer

The plasma PFOS concentrations were lower in the infant compared to maternal concentrations, while the opposite was the case for PFOA (Table 2). Both PFOS and PFOA were reduced in the mother with increasing parity: geometric means, nulliparous 6.85 ng/mL [6.72–6.98] and 1.40 ng/mL [1.37–1.43], primiparous 5.98 ng/mL [5.86–6.10] and 0.91 ng/mL [0.88–0.93], and multiparous 5.29 ng/mL [5.15–5.44] and 0.77 ng/mL [0.74–0.79], (linear effect of parity −0.13 [−0.17; −0.10] and −0.33 [−0.37; −0.29] both *p*<0.0001) for PFOS and PFOA (Figure E1), but r—squared for parity were lower for PFOS (7%) than PFOA (27%).

Infant plasma concentrations of both PFOS and PFOA were strongly associated with the number of days breastfed, and this relationship interacted statistically significant with maternal plasma concentrations of the compound in question (interaction between maternal plasma concentration and breastfeeding duration on child plasma concentration *p*<0.001) and exhibited zero order transfer kinetics (Figure E2).

Variation in the child plasma concentrations explained by maternal plasma concentration and breastfeeding showed r—squared 0.64 and 0.55 for PFOS and PFOA, respectively.

### Environmental determinants of maternal plasma concentrations

Among 675 birth addresses, 539 (80%) received drinking water from waterworks with samples analyzed for PFOS and 500 (74%) analyzed for PFOA. PFOS and PFOA were measured above detection limit of 0.001 µg/L in 0.5% and 16% of birth addresses, respectively. There was no association between maternal concentrations of either PFOS or PFOA during pregnancy and presence in drinking water samples (online Table E1).

Both maternal compounds were negatively associated with rural-urban scale, i.e. lower concentrations in women living in more urban environments. Other than this, race, maternal BMI, maternal education, and fish intake (CMPF) showed associations to maternal plasma concentrations (online Table E1).

### PFOS and PFOA exposure, intrauterine and childhood growth outcomes

**Figure 1:**
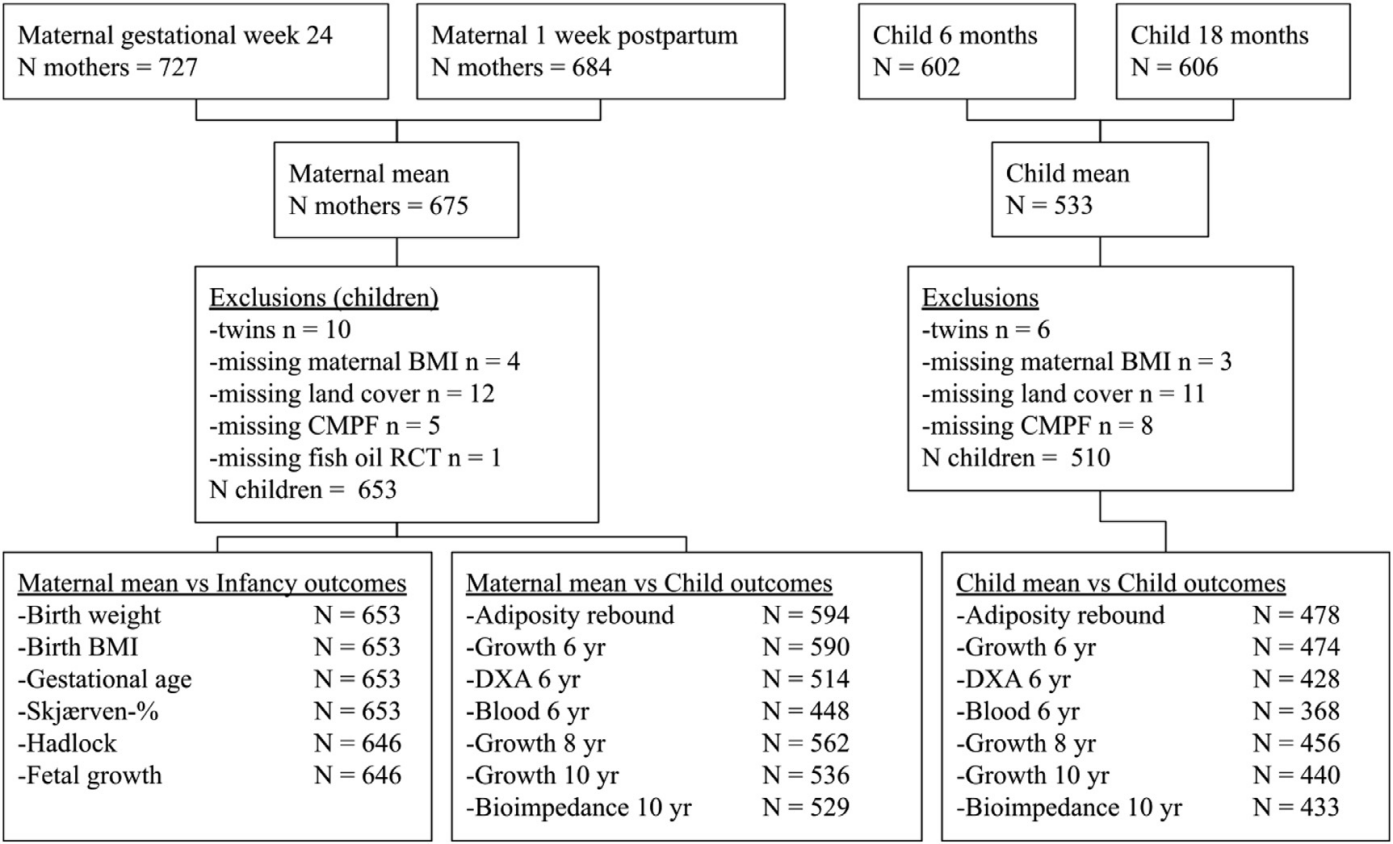
Overview of participants in analyses.

In all growth analyses twins are excluded and only children with all available data are included, see Figure 1 for overview of participants in all analyses. PFOA exposure during pregnancy was associated with a per 1-ng/mL −0.19 BMI z-score [95%CI: −0.33; −0.05] *p*=0.01, PFOS with a per 1-ng/mL −0.04 BMI z-score [95% CI: −0.08; −0.01] *p*=0.01 in confounder adjusted analyses. There were no differences in the Hadlock estimated fetal weight at pregnancy week 20, indicating late pregnancy as the vulnerable window of effect. (Table 3). When applying multipollutant models the associations diminished (online Table E2)

**Table 3:** PFOS and PFOA exposure in pregnancy and estimates for perinatal growth measures. All beta estimates (95%CI), p-value represent the effect per 1-ng/mL change in the concentration. Estimates are adjusted for parity, ethnicity, fish oil intervention, CMPF (biomarker for fish intake), maternal BMI, maternal height, maternal social circumstances and urban-rural gradient.

	*N*	PFOS	PFOA
Hadlock w 20. (ultrasound scan)	646	0.65 [−1.39; 2.70] 0.53	2.23 [−6.68; 11.14] 0.62
Fetal growth. (birth minus ultrasound scan)	646	−0.10 [−0.20; 0.00] 0.06	−0.36 [−0.80; 0.07] 0.10
BMI at birth, z-score	653	−0.04 [−0.08; −0.01] 0.01	−0.19 [−0.33; −0.05] 0.01
Gestational age at birth, days	653	−0.23 [−0.57; 0.10] 0.17	−0.62 [−2.08; 0.84] 0.41
Weight at birth, z-score	653	−0.04 [−0.07; −0.01] 0.02	−0.14 [−0.28; 0.01] 0.06
Birth weight % for sex and gestational age	653	−1.07 [−1.96; −0.19] 0.02	−4.28 [−8.17; −0.39] 0.03

Table 4 shows results on childhood growth outcomes from both maternal and infancy exposure. Increasing maternal, but not infancy, plasma PFOA concentrations associated with increasing child height: pr 1-ng/mL 0.21 higher z-score height at 10 years [95%CI: 0.07–0.36], especially in multipollutant models (0.31 [0.15; 0.47]) adjusting for concurrent concentrations of plasma PFOS (online Table E3). For PFOS there were no associations with child height for maternal concentrations, but infancy concentrations associated with lower height later in childhood.

**Table 4:** Child growth and obesity outcomes by maternal and infancy exposure to PFOS and PFOA. All beta estimates (95% CI) *p*-value represent the effect per 1-ng/mL change in the respective compound. All estimates are adjusted for parity, ethnicity, fish oil intervention, CMPF (biomarker for fish intake), maternal BMI, maternal height, maternal social circumstances and urban–rural gradient. Effects of infancy exposure is further adjusted for breastfeeding duration. Pregnancy exposure is shown as the mean of pregnancy week 24 and 1 week postpartum levels and infancy exposure as the mean of 6 and 18 month levels. Stars indicate statistical significant interaction between child sex and PFOS/PFOA.

	Pregnancy	Infancy
**PFOS**		
Adiposity rebound	0.01 [−0.04; 0.05] 0.81	0.02 [−0.03; 0.08] 0.41
DXA fat %, 6 yr	0.01 [−0.02; 0.05] 0.41 *	0.01 [−0.03; 0.05] 0.70
BMI z-score 6 yrs	−0.02 [−0.04; 0.01] 0.22 *	−0.03 [−0.06; 0.01] 0.10
BMI z-score 8 yrs	−0.01 [−0.04; 0.02] 0.58	−0.01 [−0.05; 0.02] 0.48
BMI z-score 10 yrs	−0.02 [−0.05; 0.02] 0.38	−0.02 [−0.06; 0.02] 0.33
Height z-score 6 yrs	−0.01 [−0.04; 0.02] 0.35	−0.06 [−0.10; −0.03] <0.01
Height z-score 8 yrs	−0.02 [−0.05; 0.01] 0.25	−0.06 [−0.10; −0.03] <0.01
Height z-score 10 yrs	−0.02 [−0.05; 0.01] 0.27	−0.05 [−0.09; −0.01] <0.01
BCA 10 yr FFMI	−0.04 [−0.08; 0.00] 0.05	−0.04 [−0.09; 0.01] 0.09
BCA 10 yr FMI	0.00 [−0.05; 0.05] 0.97	0.01 [−0.05; 0.07] 0.72
**PFOA**		
Adiposity rebound	−0.19 [−0.40; 0.02] 0.08	0.01 [−0.07; 0.09] 0.82
DXA fat %, 6 yr	0.13 [−0.02; 0.29] 0.09	0.01 [−0.05; 0.07] 0.67
BMI z-score 6 yrs	−0.03 [−0.15; 0.09] 0.67	−0.03 [−0.08; 0.02] 0.23
BMI z-score 8 yrs	0.04 [−0.10; 0.18] 0.55	0.00 [−0.06; 0.05] 0.89
BMI z-score 10 yrs	0.11 [−0.05; 0.27] 0.18	−0.01 [−0.07; 0.05] 0.79
Height z-score 6 yrs	0.18 [0.05; 0.31] <0.01	−0.03 [−0.08; 0.02] 0.29
Height z-score 8 yrs	0.18 [0.05; 0.31] <0.01	−0.03 [−0.08; 0.02] 0.28
Height z-score 10 yrs	0.21 [0.07; 0.36] <0.01	−0.01 [−0.07; 0.04] 0.67
BCA 10 yr FFMI	0.00 [−0.17; 0.2] 0.96	−0.01 [−0.08; 0.06] 0.86
BCA 10 yr FMI	0.18 [−0.03; 0.39] 0.09	0.01 [−0.07; 0.09] 0.75

There were no strong associations to obesity related outcomes, but pregnancy plasma PFOS concentrations interacted with child sex on 6 years BMI and fat percentage, where girls had lower BMI and fat percentage and boys higher (sex stratified results in online Table E4).

There were no associations with blood HbA1c, HDL, LDL, cholesterol or triglycerides at age 6 (data not shown).

### PFOS and PFOA exposure, plasma metabolomics profiles and LacCers

**Figure 2:**
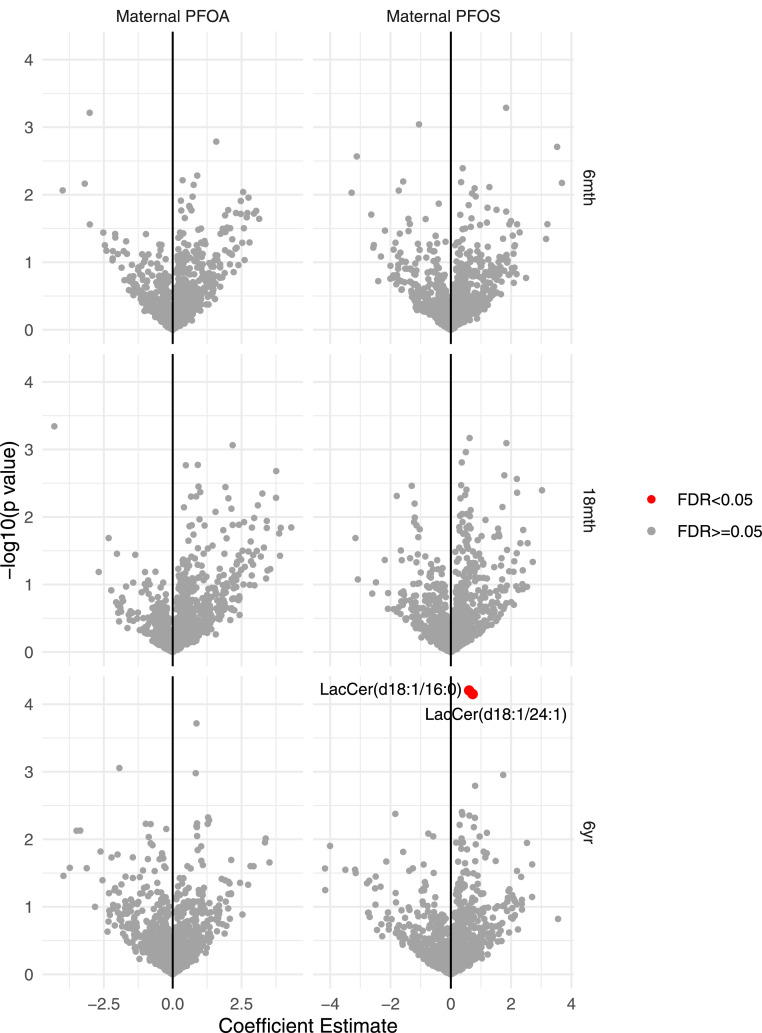
Metabolomics volcano plot showing associations between maternal PFOS and PFOA concentrations and offspring metabolome at age 6 months, 18 months and 6 years. Coefficient estimates for each metabolite are shown in the x-axis while negative log transformed *p*-values of the metabolites are plotted in the y-axis. Estimates are adjusted for breastfeeding, parity, race, social circumstances, fish oil interventions, fish intake biomarker (CMPF), maternal pre-pregnancy BMI, and urbanicity. The metabolites with positive coefficient estimates indicate positive association with maternal PFOS and PFOA concentrations.

Untargeted plasma metabolomics from the children at age 18 months and 6 years identified a total of 1305 metabolites. Of these, maternal plasma PFOS concentration was positively associated with 2 LacCer sphingolipids: LacCer(d18:1/24:1) and LacCer(d18:1/16:0) (FDR<0.05) as shown in Figure 2. Maternal plasma PFOA concentration was not associated with child plasma metabolites at any age. Associations were adjusted for breastfeeding, parity, race, social circumstances, fish oil interventions, fish intake biomarker, maternal BMI and urbanicity.

Because the untargeted explorative metabolomics approach revealed alterations in a specific group of metabolites, i.e. LacCers, we performed targeted quantification of plasma LacCer concentrations acquired at the age of 6 years. This showed that in addition to LacCer(d18:1/24:1) and LacCer(d18:1/16:0), maternal plasma PFOS concentration was also associated with 7 other LacCer species (online Table E5).

LacCers at age 6 years with statistical significant association to maternal plasma PFOS concentration were combined in a principal component analysis with PC1 explaining 69% of the variation. This component was found to borderline mediate of the negative effect of maternal plasma PFOS concentration on girl BMI at age 6 years (10% effect mediated, *p*=0.08), and borderline mediate the effect on boy fat percentage (11% effect mediated, *p*=0.07) Independently, the LacCers were associated with a later adiposity rebound, i.e. similar direction as the lower child BMI at age 6 years.

## Discussion

Concentrations of both PFOS and PFOA measured in this study were detectable in plasma of every pregnant mother and their offspring in our Danish cohort of 738 pregnant women and their children born around 2010, highlighting the ubiquitous nature of these persistent bioaccumulative compounds. This is despite that the use of PFOS has been subject to strict regulations since 2009, and PFOA since 2020 in the EU REACH regulation of chemicals.^24^ Our longitudinal exposure data showed a strong mother to child transfer of PFOS and PFOA via placenta and breastfeeding, which was dependent on maternal concentrations and parity, as shown previously.^25^^,^^26^ We found a detrimental effect on late fetal growth for both compounds. In childhood, pregnancy plasma PFOA concentrations associated with taller children, and infancy plasma PFOS concentrations associated with lower height. Pregnancy plasma PFOS concentrations interacted with child sex on childhood BMI and fat percentage at age 6 years, where girls had lower BMI and boys higher by increasing maternal concentrations. The adverse effect of pregnancy PFOS exposure on child BMI was mediated through changes in the child's metabolome with increased LacCers.

## Caveats and limitations

It is a main strength of this study that it is part of the ongoing single-center COPSAC2010 mother-child cohort study and uses meticulously collected and validated clinical data. All baseline data was collected at several time points and manually compared for consistency. All data was double-checked and locked, which minimized the risk of incorrect registration in the database. The families are followed by trained staff using predefined standard operating procedures. All clinical staff and physicians in this study have pediatric training, ensuring high quality of data collection, validation and homogenous diagnostic procedures. The cohort has low attrition and many confounding variables were collected prospectively. Still the risk of residual confounding can never be ruled out.

It is a limitation that only PFOS and PFOA were assessed. A recent report showed higher transplacental transfer of other PFAS compounds, i.e. chlorinated polyfluorinated ether sulfonates, Cl-PFESAs for PFOS and perfluorobutanoic acid, PFBA for PFOA, highlighting the need to investigate additional compounds.^27^ Furthermore, it is a limitation that the compounds were assessed through the semi-quantitative untargeted metabolomics profile, but we calibrated these values by analysing a selected panel of samples using a targeted pipeline.

## Interpretation

We showed a statistical significant association with the urbanicity scale previously associated with the infant microbiome and later development of asthma,^20^ where rural mothers had higher concentrations of PFOS and PFOA. Others have reported differences by geographic location, but not urbanicity.^28^ Also fish intake associates with maternal concentrations as shown previously.^29^ In general, the environmental factors we studied did not explain much of the variation in PFOS and PFOA exposure between mothers, and we were unable to establish any link between presence in drinking water and maternal concentrations.

Levels of PFOS and PFOA in this Danish cohort are lower than reported US levels by around 30–50% compared to NHANES 2007-8. The concentrations reported from the US were 10.7 ng/mL (9.72–11.8) for PFOS and 3.56 ng/mL (3.38–3.74) for PFOA.^30^ Child levels were also lower than a Norwegian cohort of 3 year olds,^31^ but comparable to other European pregnancy cohorts from Denmark and Spain.^8^^,^^32^ Levels are in general lower for maternal but similar for child samples (5 years vs our 18 months) in a same age Faroese cohort.^9^ Infant PFOA concentrations were statistically significant higher than maternal concentrations as opposed to PFOS, where offspring concentrations were statistically significant lower than maternal concentrations. This indicates a compound specific placental transfer for some PFAS species, where PFOA transfers three times higher than PFOS as demonstrated in other studies.^27^^,^^33^ The higher child concentrations of PFOA could also be caused by an unknown acute exposure in this region or lower accuracy of the analytical method to measure PFOA as compared to PFOS. We also showed that PFOS and PFOA in the child increased with duration of breastfeeding in agreement with previous studies,^31^^,^^34^ and that child's PFOS and PFOA levels were inversely related with the number of older siblings.^35^ This was mirrored in the mothers load of PFOS and PFOA, which was reduced with parity and also in agreement with previous research.^36^ We found that transfer of PFOS and PFOA from mother to child resulted in high infant levels, particularly in breastfed infants who thereby act as “a sink” for the mother's exposure passing these xenobiotics on for generations.^37^

Associations between pregnancy PFOS and PFOA exposure and offspring birth weight have been examined in multiple studies, showing mostly effect on female infants with lower birth weight and lower gestational age by higher prenatal PFOS and PFOA exposure.^38^, ^39^, ^40^, ^41^ Importantly, our study showed that the hampering of fetal growth by PFOS and PFOA exposure was most prominent later in pregnancy, and there were no differences in Hadlock estimated fetal weight in pregnancy week 20.

Several previous studies have found positive associations between maternal PFAS exposure and increased early childhood BMI measures,^8^^,^^32^^,^^42^, ^43^, ^44^, ^45^ which is interesting since our data did not replicate these findings, instead we showed statistically significant associations to higher height for age and sex by increasing maternal plasma PFOA concentrations, which is opposite of a large NHANES study.^46^ The PFOA—height associations did not replicate in childrens own concentrations of plasma PFOA in infancy indicating importance of exposure timing. For PFOS we found decreased height more than BMI, however, only statistically significant for infancy plasma PFOS exposure. For the association between increasing maternal plasma PFOS exposure in pregnancy and lower girl BMI at age 6 years, but higher boy fat percentage, we found support for a possible biochemical mechanism as maternal plasma PFOS was associated with alterations in the child metabolome through increasing LacCers, which in turn showed some mediation of the sex specific effects on growth from maternal plasma PFOS. In line with this, previous studies have shown association between PFOA and PFOS levels and sphingomyelins^47^^,^^48^ but to our knowledge this has not been shown for LacCers. LacCers have previously been associated with inflammation and oxidative stress,^49^ which could hamper intrauterine and childhood growth and act as a possible mechanism behind our findings. We did not replicate previous findings of perturbations in amino acid and glycerophospholipid metabolism associated with prenatal PFAS.^50^

In a recent American study with follow-up into adulthood, PFOS was associated with lower BMI throughout childhood, whilst PFOA was associated with lower BMI in early childhood, but earlier adiposity rebound and later growth.^44^^,^^51^ The latter is in line with our data. Earlier adiposity rebound appears to be associated with later obesity.^15^

Conclusively, our longitudinal mother-child cohort data on PFOS and PFOA exposure in pregnancy and early childhood showed strong transfer from mother to child with compound specific transfer so PFOA concentrations were higher in children than mothers, transfer via breastfeeding was dependent on maternal concentrations. Increasing pregnancy plasma PFOS and PFOA exposure was associated with reduced fetal growth, opposing effects on child height and sex—interacting effects on child BMI and fat percentage by PFOS, potentially acting through an altered LacCer metabolism that has been associated with inflammation and oxidative stress.

## Contributors

The guarantor of the study is BC, from conception and design to conduct of the study and acquisition of data. AS drafted the manuscript. AS, GG, DR, CETP, MAR, SSN performed analyses. JSch calculated exposure from water wells. KS and JM calibrated the semi quantitative data. AC, PZ, PW generated the targeted sphingoliphids profiles. AS and GG have verified the underlying data. AS, GG, DR, CETP, JALS, AC, PZ, CEW, SSN, DMK, MAR, JSch, KS, JWM, JS, KB, HB and BC have provided important intellectual input and contributed considerably to the analyses and interpretation of the data. All authors guarantee that the accuracy and integrity of any part of the work have been appropriately investigated and resolved and all have approved the final version of the manuscript. The corresponding author had full access to the data and had final responsibility for the decision to submit for publication. No honorarium, grant, or other form of payment was given to any of the authors to produce this manuscript.

## Data sharing statement

Privacy is important to us which is why we follow national and international legislation on General Data Protection Regulation (GDPR), the Danish Act on Processing of Personal Data and the practice of the Danish Data Inspectorate. All data that supports the findings in this study, including clinical data, are available from the corresponding author upon reasonable request: participant-level personally identifiable data are protected under the Danish Data Protection Act and European Regulation 2016/679 of the European Parliament and of the Council (GDPR) that prohibit distribution even in pseudo-anonymized form, but can be made available under a data transfer agreement as a collaboration effort.

## Declaration of interests

All authors declare no potential, perceived, or real conflict of interest regarding the content of this manuscript. No pharmaceutical company was involved in the study.

## Appendix Supplementary materials

Image, application 1
