# Supplementary material for: Effect of perfluoroalkyl exposure in pregnancy and infancy on intrauterine and childhood growth and anthropometry. Sub study from COPSAC2010 birth cohort
Source: eBioMedicine. 2022 Aug 26;83:104236. doi: 10.1016/j.ebiom.2022.104236 (PMC9434040; doi:10.1016/j.ebiom.2022.104236)
Supplement: Supplementary file 1 [file mmc1.pdf]

ONLINE SUPPLEMENT

METHODS

Batch normalisation

Batch normalisation was performed to compensate for inter-day variation. Specifically, each metabolite was corrected in run-day blocks by registering the medians to equal one (1.00) and correcting each variable accordingly. Thereafter, the datasets from all the four platforms were imported into RStudio (Version 1.1, RStudio, Inc) for statistical analysis. The maternal and child age 18 months plasma samples were analysed at a later stage compared to the child age 6 months and 6 years samples. Therefore, we used 20 replicated samples, which were analysed with the 6 months and 6 years batch (t1) and with the maternal and child age 18 months batch (t2). For each compound we calculated a ratio between t2 and t1 for each 20 replicated sample and the median of the ratios was used as a correction factor for the maternal and child age 18 months dataset.

Determination of Lactosylceramides

Samples were divided in batches of 45 samples, 2 QCext and 1 blank of extraction. For each batch, samples were thawed at 4°C and vortexed for 20 seconds. Afterwards, a volume of 50 µl of each sample was aliquoted on an Eppendorf tube. Then, 20 µl of an internal standard mixture, containing d_3-Lactosylceramide(d18:1/16:0) (Matreya; PN 1534) was added to each sample. Tubes were then vortexed for 10 seconds and allowed to equilibrate with the internal standard for 10 minutes at 20°C. Then, a volume of 500 µl of methanol was added to each sample. Eppendorf tubes were then closed and vortexed for 10 seconds. Afterwards, samples were sonicated for 15 minutes on an ice bath to avoid the temperature increasing above 20°C. Samples were then centrifuged at 12000 *g* for 15 minutes. An aliquot of 80 µl was finally transferred to an LC-MS vials equipped with a 150 µl insert. To prepare the QCinj (1 injected every 14 samples), 20 µl of each sample was pooled into a 4 mL glass vial. All samples and QCs were stored at -20°C until the day of analysis (within 3 days after extraction). Chromatographic separation was carried out on an ACQUITY UPLC System with a sample manager cooled to 8°C (both from Waters Corporation, Milford, MA, USA). Lactosylceramides were separated on a Zorbax Rapid Resolution RRHD C18 Column, 80Å, 1.8 µm, 2.1 mm X 100 mm (Agilent Technologies; Product Number: 758700-902) using a guard column (Agilent Technologies, PN: 821725-901) (5 × 2 mm, 1.8 µm particle size).

Mobiles phases A an B consisted of 5mM ammonium formate (Sigma; PN: 70221) / 0.2% formic acid (Optima, Fisher-Scientific, PN: 10596814) in water and in methanol (VWR, PN: 34966), respectively. Separation was carried out at a 450 µl/min flow rate and column temperature was held at 40°C. The following chromatographic gradient was used: 0 min, 75% B; time range 0 → 1 min, 75% B (constant); time range 1 → 5 min, 85 → 100% B (linear increase); time range 5 to 15.2 min, 100% B (isocratic range); time range 15.2 → 15.3 min, 100 → 75% B (linear decrease); time range 15.3 → 16 min, 75% B (isocratic column conditioning). Samples were then analysed on a Waters Xevo® TQ-S system equipped with an Electrospray Ion Source (ESI) and ScanWave™ collision cell technology operating in the positive mode. An specific single reaction monitoring (SRM) transition for each lactosylceramide and internal standard was used. For compounds reported with full quantification, an external calibration curve was built for each compound using external standards (Avanti Lipids, PN 860545, 860576, 860597, 860577, 860590 and 860598). For compounds with pseudoquantification, the retention time was predicted based on the number of carbons and unsaturations, relative to the compounds quantified with a standard. A pseudo-calibration curve was then built using the closer eluting compound.

Land cover and Detection of PFAS in public water systems

Birth address of participants was obtained at interviews. Via a public database with all Danish addresses (https://dawadocs.dataforsyningen.dk/), the longitude and latitude were obtained. These were linked to other publicly available data for land use classification (https://land.copernicus.eu/pan-european/corine-land-cover).

Similarly each household address at birth was assigned to the specific waterwork it was supplied by. PFOS and PFOA in drinking water samples were measured by certified laboratories and registered in the national monitoring database Jupiter, from which they were extracted, quality-controlled and collated to “never measured for PFOS/PFOA”, “never detected above detection limit”; and “at least one sample above detection limit”. For both PFOS and PFOA, the highest ever amount measured at the respective waterworks was calculated. Most tests for PFOS and PFOA were done after 2018, so levels at birth were extrapolated.

ONLINE TABLES & FIGURES

Figure E1

Maternal (N=675) pregnancy plasma PFOS and PFOA concentrations shown as the mean of pregnancy week 24 and 1 week postpartum measurement by parity.

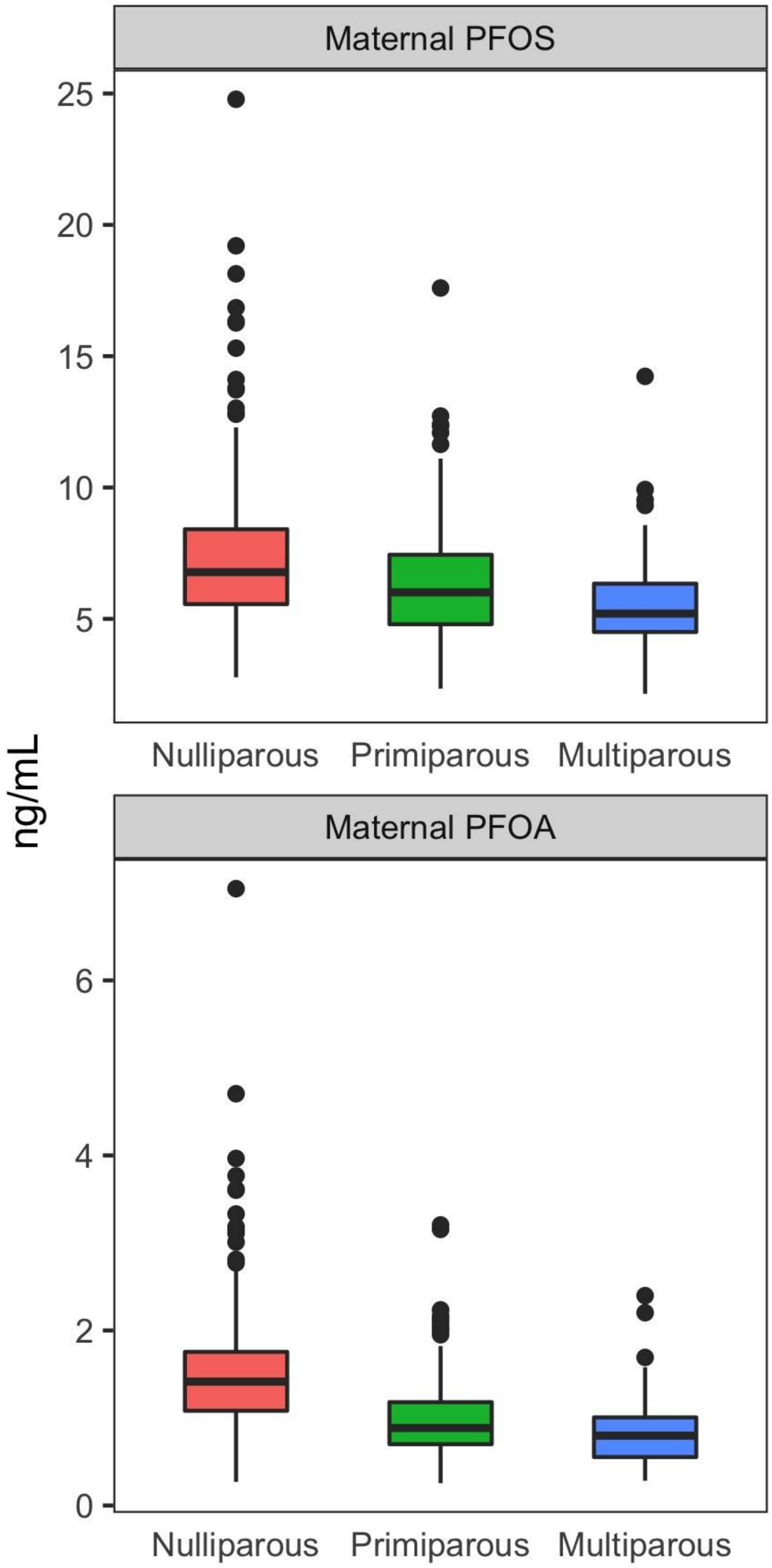
Maternal PFOS
25
20
15
10
5
Nulliparous
Primiparous
Multiparous
ng/mL
Maternal PFOA
6
4
2
0
Nulliparous
Primiparous
Multiparous

70

Figure E2

Child (N=521) mean PFOS and PFOA concentrations shown against days of breastfeeding and maternal concentrations in tertiles. Numbers are offset with 0.8 for PFOA due to negative extrapolated values from the quantification.

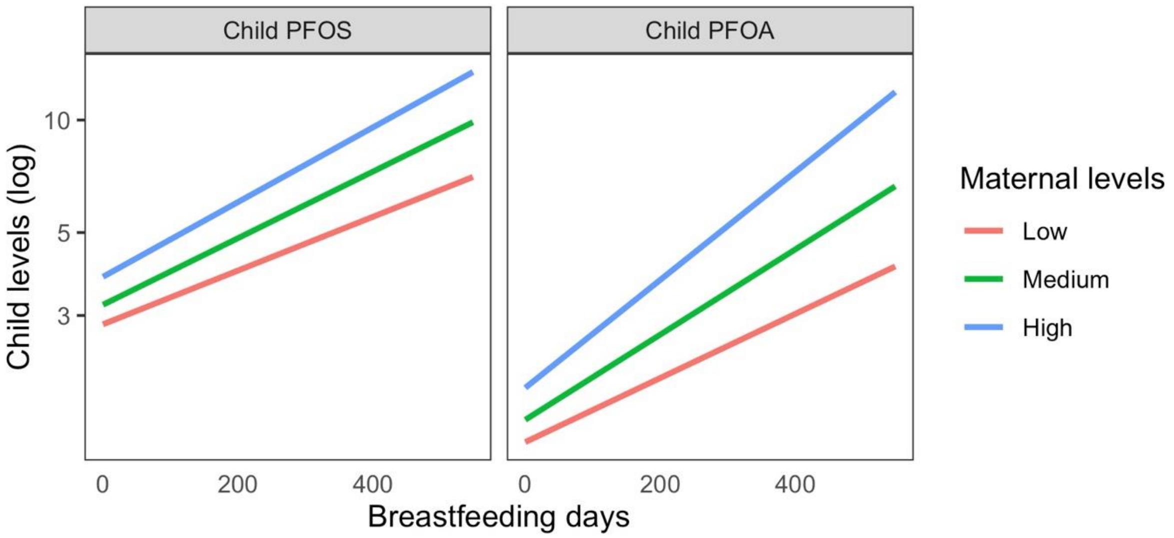

Online Table E1.

Associations to maternal concentrations of PFOS and PFOA. Estimates interpretes as percentage change in geometric mean, numeric predictors are z-scored, land cover uses scaled variable.

	**PFOS: estimate, p (t-statistic)**	**PFOA: estimate, p (t-statistic)**
Maternal age	-1%, 0.30	-8%, <0.01
Maternal BMI	-1%, 0.47	2%, 0.20
Maternal smoking	1%, 0.94	6%, 0.54
Maternal FLG risk*	-1%, 0.86	13%, 0.18
Fish intake biomarker cmpf	6%, <0.01	4%, 0.05
Maternal education	-2%, 0.43	-5%, 0.18
Family income	-1%, 0.56	-3%, 0.03
Parity	-12%, <0.01	-28%, <0.01
Drinking water >LoD	14%, 0.50	-4%, 0.39
Land cover gradient	-4%, 0.91	-186%, 0.04
Race, european descent	-13%, 0.03	-7%, 0.44
Fishoil intervention group	-1%, 0.62	4%, 0.24

*Maternal filaggrin risk is based on SNPs: rs138726443; rs150597413; rs61816761.

Online Table E2

PFOS and PFOA exposure in pregnancy and estimates for perinatal growth measures. All beta estimates (95%CI), p-value represent the effect per 1-ng/mL change in the concentration. Estimates are adjusted for parity, ethnicity, fish oil intervention, CMPF (biomarker for fish intake), maternal BMI, maternal height, maternal social circumstances and urban-rural gradient, **plus adjustment for the other PFAS**.

	PFOS	PFOA
Hadlock w 20. (ultrasound scan)	0.55 [-1.77; 2.87] 0.64	1.15 [-8.79; 11.10] 0.82
Fetal growth. (birth minus ultrasound scan)	-0.07 [-0.18; 0.05] 0.24	-0.23 [-0.71; 0.26] 0.36
BMI at birth, z-score	-0.03 [-0.06; 0.01] 0.16	-0.14 [-0.30; 0.02] 0.09
Gestational age at birth, days	-0.20 [-0.57; 0.18] 0.31	-0.17 [-1.80; 1.46] 0.83
Weight at birth, z-score	-0.03 [-0.07; 0.01] 0.14	-0.08 [-0.24; 0.08] 0.35
Birth weight % for sex and gestational age	-0.72 [-1.72; 0.28] 0.16	-2.79 [-7.13; 1.54] 0.21

Online Table E3

Multipollutant models.

Child growth and obesity outcomes by maternal and infancy exposure to PFOS and PFOA. All beta estimates (95% CI) p-value represent the effect per 1-ng/mL change in the respective compound. All estimates are adjusted for parity, ethnicity, fish oil intervention, CMPF (biomarker for fish intake), maternal BMI, maternal height, maternal social circumstances and urban-rural gradient, **and the other PFAS**. Effects of infancy exposure is further adjusted for breastfeeding duration. Pregnancy exposure is shown as the mean of pregnancy week 24 and 1 week postpartum levels and infancy exposure as the mean of 6 and 18 month levels.

	**Pregnancy**	**Infancy**
**PFOS**		
Adiposity rebound	0.03 [-0.02; 0.09] 0.24	0.03 [-0.04; 0.10] 0.41
DXA fat %, 6 yr	0.00 [-0.04; 0.04] 0.98 *	0.00 [-0.04; 0.05] 0.83
BMI z-score 6 yrs	-0.02 [-0.05; 0.01] 0.26 *	-0.02 [-0.06; 0.01] 0.24
BMI z-score 8 yrs	-0.02 [-0.05; 0.02] 0.36	-0.02 [-0.06; 0.03] 0.46
BMI z-score 10 yrs	-0.03 [-0.08; 0.01] 0.09	-0.02 [-0.07; 0.02] 0.33
Height z-score 6 yrs	-0.04 [-0.08; -0.01] 0.01	-0.07 [-0.11; -0.03] <0.01
Height z-score 8 yrs	-0.05 [-0.08; -0.01] <0.01	-0.07 [-0.11; -0.03] <0.01
Height z-score 10 yrs	-0.05 [-0.09; -0.02] <0.01	-0.06 [-0.11; -0.02] <0.01
BCA 10 yr FFMI	-0.05 [-0.10; -0.01] 0.02	-0.05 [-0.11; 0.00] 0.06
BCA 10 yr FMI	-0.02 [-0.08; 0.03] 0.38	0.01 [-0.06; 0.07] 0.82
**PFOA**		
Adiposity rebound	-0.26 [-0.50; -0.02] 0.03	-0.01 [-0.11; 0.09] 0.83
DXA fat %, 6 yr	0.14 [-0.03; 0.31] 0.12	0.01 [-0.06; 0.08] 0.79
BMI z-score 6 yrs	0.02 [-0.12; 0.15] 0.82	-0.01 [-0.07; 0.04] 0.60
BMI z-score 8 yrs	0.08 [-0.07; 0.23] 0.31	0.01 [-0.05; 0.07] 0.82
BMI z-score 10 yrs	0.18 [0.00; 0.36] 0.05	0.01 [-0.06; 0.08] 0.81
Height z-score 6 yrs	0.25 [0.11; 0.40] <0.01	0.02 [-0.04; 0.07] 0.59
Height z-score 8 yrs	0.26 [0.12; 0.41] <0.01	0.02 [-0.04; 0.08] 0.54
Height z-score 10 yrs	0.31 [0.15; 0.47] <0.01	0.03 [-0.03; 0.10] 0.30
BCA 10 yr FFMI	0.11 [-0.09; 0.32] 0.28	0.03 [-0.05; 0.11] 0.45
BCA 10 yr FMI	0.23 [0.00; 0.46] 0.05	0.01 [-0.09; 0.10] 0.87

Online Table E4.

Sex stratified growth outcomes. All beta estimates (95% CI, and t-statistic p-values) represent the effect per 1-ng/mL change in the respective compound. All estimates are adjusted for parity, ethnicity, fish oil intervention, CMPF (biomarker for fish intake), maternal BMI, maternal social circumstances and urban-rural gradient. Infancy additionally adjusted for breastfeeding duration. Columns with multipollutant models further adjust for concentration of the other compound. Pregnancy exposure is shown as the mean of pregnancy week 24 and 1 week postpartum levels and infancy exposure as the mean of 6 and 18 month levels.

	Pregnancy	Pregnancy, multipollutant	Infancy	Infancy, multipollutant
**Females: PFOS**				
Adiposity rebound	0.01 [-0.07; 0.09] 0.83	0.04 [-0.05; 0.13] 0.35	-0.04 [-0.15; 0.06] 0.39	-0.05 [-0.17; 0.06] 0.37
DXA fat %, 6 yr	-0.04 [-0.09; 0] 0.07	-0.07 [-0.12; -0.02] 0.0063	-0.05 [-0.11; 0.01] 0.079	-0.06 [-0.12; 0] 0.057
BMI z 6 yrs	-0.06 [-0.1; -0.02] 0.0068	-0.06 [-0.11; -0.01] 0.012	-0.03 [-0.08; 0.02] 0.18	-0.03 [-0.09; 0.02] 0.25
BMI z 8 yrs	-0.04 [-0.09; 0.01] 0.12	-0.05 [-0.1; 0] 0.060	-0.01 [-0.07; 0.05] 0.77	-0.01 [-0.08; 0.06] 0.75
BMI z 10 yrs	-0.04 [-0.1; 0.02] 0.16	-0.06 [-0.12; 0] 0.070	-0.01 [-0.08; 0.06] 0.76	-0.01 [-0.09; 0.07] 0.84
Height z 6 yrs	-0.04 [-0.08; 0.01] 0.14	-0.05 [-0.11; 0] 0.044	-0.07 [-0.13; -0.01] 0.019	-0.08 [-0.14; -0.01] 0.027
Height z 8 yrs	-0.05 [-0.1; 0] 0.05	-0.07 [-0.12; -0.02] 0.011	-0.09 [-0.15; -0.03] 0.0059	-0.09 [-0.16; -0.02] 0.013
Height z 10 yrs	-0.05 [-0.11; 0.01] 0.09	-0.07 [-0.14; -0.01] 0.024	-0.08 [-0.15; -0.01] 0.036	-0.08 [-0.16; 0] 0.059
BCA 10 yr FFMI	-0.07 [-0.14; -0.01] 0.031	-0.08 [-0.16; -0.01] 0.025	-0.01 [-0.09; 0.07] 0.78	-0.01 [-0.1; 0.08] 0.76
BCA 10 yr FMI	-0.06 [-0.13; 0.02] 0.13	-0.09 [-0.17; -0.01] 0.029	0 [-0.09; 0.09] 0.99	0 [-0.11; 0.1] 0.96
**Males: PFOS**				
Adiposity rebound	0.01 [-0.05; 0.07] 0.65	0.03 [-0.04; 0.1] 0.40	0.06 [-0.01; 0.13] 0.073	0.08 [0; 0.16] 0.050
DXA fat %, 6 yr	0.06 [0.02; 0.1] 0.0047	0.06 [0.01; 0.11] 0.011	0.03 [-0.01; 0.08] 0.17	0.04 [-0.01; 0.1] 0.11
BMI z 6 yrs	0.02 [-0.02; 0.05] 0.41	0.01 [-0.03; 0.06] 0.56	-0.01 [-0.06; 0.03] 0.50	-0.01 [-0.06; 0.04] 0.72
BMI z 8 yrs	0.01 [-0.03; 0.05] 0.63	0.01 [-0.04; 0.06] 0.75	-0.01 [-0.06; 0.04] 0.65	-0.02 [-0.07; 0.04] 0.52
BMI z 10 yrs	0 [-0.04; 0.05] 0.89	-0.02 [-0.07; 0.04] 0.55	-0.01 [-0.07; 0.04] 0.63	-0.03 [-0.09; 0.03] 0.40
Height z 6 yrs	0 [-0.04; 0.05] 0.84	-0.03 [-0.08; 0.02] 0.28	-0.05 [-0.11; 0] 0.048	-0.05 [-0.11; 0.01] 0.11
Height z 8 yrs	0.01 [-0.04; 0.06] 0.72	-0.02 [-0.08; 0.03] 0.44	-0.05 [-0.1; 0.01] 0.090	-0.05 [-0.11; 0.02] 0.15
Height z 10 yrs	0 [-0.04; 0.05] 0.91	-0.03 [-0.09; 0.02] 0.27	-0.04 [-0.09; 0.01] 0.16	-0.04 [-0.11; 0.02] 0.16
BCA 10 yr FFMI	-0.01 [-0.06; 0.04] 0.67	-0.02 [-0.08; 0.04] 0.51	-0.03 [-0.08; 0.03] 0.32	-0.05 [-0.11; 0.01] 0.13
BCA 10 yr FMI	0.04 [-0.02; 0.1] 0.19	0.03 [-0.04; 0.1] 0.44	0.01 [-0.05; 0.08] 0.71	0.01 [-0.07; 0.09] 0.80

**Females: PFOA**				
Adiposity rebound	-0.25 [-0.57; 0.06] 0.11	-0.31 [-0.65; 0.03] 0.072	-0.01 [-0.15; 0.13] 0.88	0.02 [-0.13; 0.18] 0.78
DXA fat %, 6 yr	0.14 [-0.04; 0.32] 0.13	0.22 [0.03; 0.42] 0.022	0 [-0.08; 0.08] 0.94	0.03 [-0.05; 0.12] 0.46
BMI z 6 yrs	-0.08 [-0.25; 0.08] 0.34	0 [-0.18; 0.17] 0.99	-0.02 [-0.09; 0.04] 0.48	0 [-0.08; 0.07] 0.91
BMI z 8 yrs	0.04 [-0.14; 0.23] 0.64	0.11 [-0.09; 0.31] 0.27	0 [-0.08; 0.08] 0.99	0.01 [-0.08; 0.1] 0.89
BMI z 10 yrs	0.08 [-0.14; 0.3] 0.46	0.15 [-0.08; 0.39] 0.20	-0.01 [-0.11; 0.08] 0.78	-0.01 [-0.12; 0.1] 0.87
Height z 6 yrs	0.08 [-0.1; 0.27] 0.39	0.15 [-0.05; 0.35] 0.14	-0.03 [-0.12; 0.05] 0.42	0.01 [-0.08; 0.1] 0.79
Height z 8 yrs	0.09 [-0.1; 0.29] 0.35	0.19 [-0.02; 0.39] 0.076	-0.05 [-0.14; 0.03] 0.24	0 [-0.1; 0.09] 0.99
Height z 10 yrs	0.11 [-0.11; 0.34] 0.33	0.2 [-0.04; 0.44] 0.098	-0.05 [-0.15; 0.05] 0.36	0 [-0.11; 0.11] 0.98
BCA 10 yr FFMI	0 [-0.27; 0.26] 0.99	0.11 [-0.17; 0.39] 0.45	0 [-0.12; 0.12] 0.99	0.01 [-0.12; 0.14] 0.90
BCA 10 yr FMI	0.17 [-0.12; 0.46] 0.25	0.28 [-0.03; 0.58] 0.076	0.01 [-0.12; 0.14] 0.91	0.01 [-0.14; 0.16] 0.90
**Males: PFOA**				
Adiposity rebound	-0.07 [-0.35; 0.22] 0.65	-0.18 [-0.52; 0.16] 0.30	0.01 [-0.1; 0.11] 0.87	-0.05 [-0.17; 0.07] 0.42
DXA fat %, 6 yr	0.13 [-0.07; 0.33] 0.20	-0.01 [-0.25; 0.23] 0.95	0 [-0.07; 0.07] 0.95	-0.04 [-0.12; 0.04] 0.38
BMI z 6 yrs	0.06 [-0.12; 0.23] 0.54	0.04 [-0.17; 0.25] 0.71	-0.03 [-0.09; 0.04] 0.44	-0.02 [-0.09; 0.05] 0.60
BMI z 8 yrs	0.04 [-0.16; 0.25] 0.69	0.04 [-0.2; 0.28] 0.73	0.01 [-0.06; 0.08] 0.83	0.02 [-0.06; 0.1] 0.62
BMI z 10 yrs	0.14 [-0.09; 0.37] 0.24	0.2 [-0.07; 0.47] 0.15	0.02 [-0.06; 0.1] 0.62	0.04 [-0.05; 0.13] 0.40
Height z 6 yrs	0.23 [0.01; 0.45] 0.042	0.31 [0.05; 0.57] 0.019	-0.05 [-0.13; 0.03] 0.24	-0.01 [-0.11; 0.08] 0.75
Height z 8 yrs	0.22 [-0.01; 0.45] 0.057	0.29 [0.02; 0.56] 0.036	-0.04 [-0.12; 0.05] 0.39	0 [-0.1; 0.09] 0.96
Height z 10 yrs	0.22 [0; 0.45] 0.055	0.31 [0.04; 0.58] 0.023	-0.02 [-0.1; 0.06] 0.69	0.02 [-0.08; 0.11] 0.72
BCA 10 yr FFMI	0.03 [-0.21; 0.26] 0.83	0.09 [-0.2; 0.37] 0.55	0.02 [-0.06; 0.11] 0.57	0.06 [-0.03; 0.16] 0.20
BCA 10 yr FMI	0.18 [-0.1; 0.46] 0.21	0.14 [-0.2; 0.48] 0.42	0.02 [-0.08; 0.11] 0.76	0.01 [-0.11; 0.12] 0.90

Online Table E5.

Relationship between maternal PFOS and 6 years plasma LacCers. Estimates are adjusted for parity, ethnicity, fish oil intervention, CMPF (biomarker for fish intake), maternal BMI, maternal social circumstances, urban-rural gradient and breast feeding.

	Estimate	95% CI	P.value
LacCer(d18:1/24:1)	430.8	[177.79 683.81]	0.001
LacCer(d18:1/16:0)	1189.7	[440.2 1939.14]	0.002
LacCer(d18:1/18:1)	13.4	[4.47 22.41]	0.003
*LacCer(d18:1/22:0)	89.1	[28.67 149.59]	0.004
LacCer(d18:1/18:0)	17.1	[4.39 29.74]	0.008
*LacCer(d18:1/20:0)	8.3	[1.63 15.02]	0.015
LacCer(d18:1/24:0)	67.0	[12.15 121.84]	0.017
LacCer(d18:1/17:0)	24.0	[0.91 47.17]	0.042
*LacCer(d18:1/14:0)	14.3	[-11.39 40]	0.275
*LacCer(d18:1/12:0)	1.1	[-3.32 5.57]	0.619

*indicates that the LacCer species was pseudo-quantified
